# Diagnostic Accuracy of Leucocyte Esterase Reagent Strips for Spontaneous Bacterial Peritonitis in Cirrhosis With Ascites

**DOI:** 10.1002/hsr2.73140

**Published:** 2026-08-30

**Authors:** Salum Ali Mwinyi, Emmanuel Marcel Sindato, Emmanuel Muhimbura, Gerald Jacob Kawishe, James Joseph Yahaya

**Affiliations:** ^1^ Department of Internal Medicine Benjamin Mkapa Hospital Dodoma Tanzania; ^2^ Department of Internal Medicine, School of Medicine and Dentistry University of Dodoma Dodoma Tanzania; ^3^ Department of Pathology Mubende Regional Referral Hospital Mubende Uganda; ^4^ Department of Pathology, Faculty of Medicine FINS Medical University Fort Portal Uganda; ^5^ Department of Molecular Biology and Biotechnology University of Dar‐es‐salaam Dar‐es‐salaam Tanzania; ^6^ Department of Pathology, School of Health Sciences Soroti University Soroti Uganda

**Keywords:** diagnostic accuracy, leucocyte esterase reagent strip, Liver cirrhosis, spontaneous bacterial peritonitis

## Abstract

**Background and Aims:**

Spontaneous bacterial peritonitis (SBP) in patients with liver cirrhosis and ascites is a common and serious problem which is associated with high morbidity and mortality. The present study aimed to determine the diagnostic performance of the leucocyte esterase reagent strip (LERS) for the diagnosis of SBP, and we also assessed the prevalence of SBP.

**Methods:**

This was a diagnostic accuracy study which was conducted at referral hospitals in Dodoma region, Tanzania, among patients with liver cirrhosis and ascites who were aged ≥ 18 years. Diagnostic performance of LERS was assessed by calculating the diagnostic accuracy, sensitivity, specificity, positive predictive value (PPV), and negative predictive value (NPV) at three colorimetric scales (1+, 2+, and 3+) which were analyzed as separate categories. Ascitic fluid PMN count ≥ 250 cells/mm^3^ was used as the reference standard, which is a pre‐specified diagnostic threshold.

**Results:**

The present study included a total of 269 study subjects with a mean age of 46.2 ± 10.3 years, and the majority of the patients (61.3%, *n* = 165) were males. The prevalence of SBP based on the gold standard test was (19.0%, *n* = 51). The diagnostic performance values including, accuracy, sensitivity, specificity, PPV, and NPV of LERS using CYBOWTM series urine reagent strips were considered at colorimetric scale of 2+ as comparable to the reference standard. Under this comparable point (colorimetric scale 2+), the sensitivity, specificity, PPV, and NPV were 80.4% (95% CI = 67.6–89.8), 99.1% (95% CI = 95.7–99.9), 95.3% (95% CI = 84.2–99.4), and 95.6% (95% CI = 91.9–97.9), respectively. The diagnostic accuracy of LERS at a colorimetric scale of 2+ in this study was 96.3% (95% CI = 92.3–97.6).

**Conclusions:**

This study has shown a significant proportion of SBP among the patients who were diagnosed with liver cirrhosis and had ascites. The level of diagnostic accuracy shown by LERS for SBP indicates that LERS may be used as a possible adjunctive screening tool rather than a definitive substitute for standard diagnostic assessment, especially in remote areas of resource‐limited settings where diagnostic tests like culture are still challenging.

## Summary

- Spontaneous bacterial peritonitis among cirrhotic patients is a major threat, especially in developing countries.
- Challenges in the detection of spontaneous bacterial peritonitis among cirrhotic patients contribute to high morbidity and mortality.
- Use of leucocyte esterase reagent strips for screening spontaneous bacterial peritonitis aids early detection.
- Assessment of the diagnostic accuracy, sensitivity, and specificity of the used strips warrants reliable results.

## 1 Introduction

Spontaneous bacterial peritonitis (SBP) is a severe and common complication of decompensated liver cirrhosis that has a substantial impact on quality of life and increased mortality [1, 2]. SBP is one of the most common infections among patients with liver cirrhosis, and it has almost four‐folds increased risk of occurring as compared to other infections [3, 4, 5]. The incidence of SBP in the patients with liver cirrhosis ranges from 10% to 30% globally [6, 7]. However, the burden of SBP in sub‐Saharan Africa (SSA) is much higher than that of high‐income countries (HICs) with the prevalence of SBP in such countries has been reported to be ranging from 12.5% to 67.7% [8, 9, 10]. The difference in the SBP burden between HICs and low‐and middle‐income countries (LMICs) is attributed to early diagnosis of SBP and effective use of prophylactic antibiotics, which are not affordable for most of patients in LMICs [11]. Failure or delay in diagnosing and treating SBP has been shown to be associated with poor clinical outcomes, with every hour of delay, is associated with a 3.3% rise in hospitalization [12]. Delay of diagnosis and treatment of SBP among cirrhotic patients with ascites is considered to be a common challenge among health practitioners, particularly in LMICs, due to limited diagnostic tools, costly investigations, and scarcity of trained personnel [13, 14].

Increasing prevalence of liver cirrhosis in SSA is red flag which necessitates for having readily available, cheap and rapid diagnostic tools of SBP [15]. Leucocyte esterase reagent strips (LERS) have been reported to be valuable in making rapid diagnosis of SBP among cirrhotic patients with high sensitivity, specificity, positive predictive value (PPV), and negative predictive value (NPV) [16, 17]. For example, Honar et al. reported 87.8% and 91.7% of sensitivity and specificity of LERS, respectively, among cirrhotic patients with ascites in a study which was done in Iran. In another study, which was done in India, the sensitivity, specificity, PPV, and NPV were found to be 91.3%, 90.8%, 70%, 97.5%, and 90%, respectively [18]. Gomaa et al. also reported high sensitivity and specificity of 94.6% and 100% of LERS in the diagnosis of SBP among cirrhotic patients with ascites [19]. Despite some slight variation of the diagnostic accuracy of LERS observed in different studies, the findings show that LERS can provide rapid results which can be used to initiate treatment of patients with SBP while waiting for confirmation with other diagnostic tests like culture.

Despite evidence of the diagnostic accuracy, which is rapid and less demanding, data regarding the clinical utility of LERS in Tanzania are lacking. This affects the expedition of treatment of cirrhotic patients with ascites harboring SBP at the same time, which increases hospitalization, as well as contributing to morbidity and mortality. The aim of this study was to determine the diagnostic performance of LERS by comparing it with a pre‐specified diagnostic threshold of ascitic fluid PMN count ≥ 250 cells/mm^3^, which was used as the reference standard for SBP diagnosis. Secondly, the study assessed the magnitude of SBP among patients with liver cirrhosis and ascites.

## 2 Materials and Methods

### 2.1 Study Design and Setting

This was a diagnostic accuracy study which was conducted at Benjamin Mkapa Hospital (BMH) and Dodoma Referral Hospital (DRRH) in Dodoma city, Tanzania from November 2022 to June 2023. Both BMH and DRRH are public health facilities with bed capacities of 400 and 420, respectively. These hospitals are the referral hospitals for the Dodoma region and the central zone at large. Additionally, they serve as teaching hospitals for the University of Dodoma. The population of the region showed a drastic increase of 32.5% for a period of 10 years, from 2,083,588 according to the 2012 report to 3,085,625 according to the Population and Housing Census of the United Republic of Tanzania of 2012 [20] and 2022 [21], respectively.

### 2.2 Study Population

This study included patients who were aged 18 years or over with liver cirrhosis as detected using either abdominal ultrasound or abdominal CT scan. The inclusion criteria included being 18 years or over, being diagnosed with liver cirrhosis, and having ascites. Ascites was the necessary inclusion criteria for the study subjects for indicating that they were in decompensated stage of liver cirrhosis, and this ensured homogeneity of the study population. On the other hand, all patients with secondary bacterial peritonitis, being on antibiotics for more than 12 h prior recruitment, having a history of abdominal surgery for the last 4 weeks, and having peritoneal malignancy were excluded from the analysis.

### 2.3 Sample Size Estimation and Sampling Procedure

The sample size was estimated using a formula for calculating sample size involving sensitivity assessment, which was used previously [22].

*n* = Z^2^ 1‐a/2 × Sn × (1‐Sn)

L^2^ × Prevalence

Where *n* = required sample size, Sn = expected sensitivity, L = maximum clinically acceptable width of 95% CI, α = size of the critical region (1 − α is the confidence level), Z1−α/2 = standard normal variate corresponding to the specified size of the critical region (α), and standard normal variate at 5% type 1 error (*p* < 0.05) is 1.96. Therefore, assuming standard normal variate of 1.96, maximum clinically acceptable width of 10%, and expected proportion of SBP of 17.7% as from a study done in Cameroon [23], and sensitivity of the test 95% [24]. The calculated sample size was 224. A non‐response rate of 20% was then considered to make the total sample size to be 269. Sampling of the patients in the study was done using a convenience method, in which all participants who met the inclusion criteria were enrolled consecutively.

### 2.4 Data Collection and Research Tool

All study participants were explained regarding objective of the study and its related procedures including investigations. Written informed consent was obtained prior data collection, and privacy of the patients was observed throughout the study period by anonymizing identification of the patients. Data were recruited by the researchers using a self‐made structured form. The form was prepared based on the available literature. Validation of the research tool was done after conducting a pilot study among 20 participants to obtain correctness and relevance of the items to be included in the tool. Reliability testing was done using content validity in which independent experts were involved in the assessment of the content and comprehensiveness of the research tool.

The tool comprised of five parts including sociodemographic characteristics (age of the patient, sex, and residence), clinical characteristics (abdominal pain, upper gastrointestinal bleeding, rebound tenderness, hepatic encephalopathy, fever, blood pressure, imaging findings, laboratory investigations (white blood cell count [WBC], haemoglobin [Hb] level, international normalized ratio [INR], bilirubin level, albumin level, serum creatinine level, model for end‐stage of liver disease score, and Child Pugh score), and outcome variable (presence or absence of SBP) as measured by LERS and then PMN cell count by cytology.

### 2.5 Laboratory Procedures

#### 2.5.1 Complete Blood Count Analysis

Aseptically, 10 mL of whole blood was withdrawn from the cubital fossa area of every patient, and the collected blood sample was used for different tests. Blood sampled in pink capped tubes for complete blood count (CBC) for testing of WBC, Hb level, and platelet count was done for each patient using a well calibrated automated haematology analyzer (XN 2000 SYSMEX machine, USA).

#### 2.5.2 Liver Enzymes, Serum Creatinine, and Other Blood Analyses

Part of the blood sample collected from the 10 mL was placed in red capped tubes and then used in testing for serum creatinine, albumin level, total bilirubin, and INR, and liver enzymes included alanine transaminase (ALT) aspartate transaminase (AST). A clinical chemistry automatic analyzer (Erba machine XL‐180, model 160239, Germany) was used for the chemistry analyses.

#### 2.5.3 Serological Testing of HBV and HCV

For the serological test of hepatitis B virus (HBV) and hepatitis C virus (HCV), a finger‐prick blood sample was collected aseptically from each participant. Testing of HCV infection was done by using iCARE One Step Anti‐HCV Rapid Test (Jal Medical, Singapore). A sterile plastic dropper pipette was used to draw sample from the pricked site and one drop (30 μL) of blood was added to the sample well of the test card, then two drops (80 μL) of sample diluent was added into the same well. The results were interpreted at 15 min. A purplish test band in the region was read as positive result and absence of a purplish test band in the test region was read as negative result, and there should be always a purplish control band in the control region regardless of the result, the test was considered invalid when the control band was not seen and was repeated using a new test. For qualitative detection of HBV infection; iCARE one step HBsAg Rapid Test (Jal Medical, Singapore) which is an immunochromatography assay was used. A blood sample from the pricked site was collected by means of a plastic micropipette. The micropipette was held vertically and 2 drops of blood sample were introduced to the sample well of the testing cassette. Thereafter, 2 drops of a diluent was added onto the same sample well and results were read after every 15 min. Positive results were recorded when 2 colored bands appeared as control and test lines from the testing cassette. Negative results were confirmed when only one colored band appeared for the control line, and the test was regarded as invalid if no colored band appeared for the control line.

#### 2.5.4 Leucocyte Esterase Dipstick Testing for Diagnosis of SBP

All patients underwent abdominal paracentesis, and the ascitic fluid was be aspirated in the flanks, about 3–5 cm superior and medial to the anterior superior iliac spine. The skin at the chosen point was cleaned using 70% alcohol, then ascitic fluid was withdrawn using an 18‐gauge cannula. Of the collected 20 mL of the ascitic fluid, 10 mL of the fluid was used for LERS testing. The ascitic fluid was placed in a dry and clean container that is normally used for the collection of urine. CYBOW series urine reagent strips (DFI, Gyeongsangnam‐do 50875, South Korea) were used to determine the activity of LERS. The strip was taken from the closed canister and was used immediately, and then closed tightly instantly. The reagent areas of the strip were immersed in a fresh well‐mixed ascitic fluid and were immediately removed to avoid dissolving reagents. The edge of the strip was run against the rim of the container while removing it to remove excess ascitic fluid. The strip was held in a horizontal position and the edge of the strip was brought in contact with a paper towel to avoid mixing chemicals from the adjacent reagent area and/or soiling hands with ascitic fluid. The reagent was then compared to the corresponding color blocks on the canister label after 90 s. Each reading was graded by two well‐trained medical personnel who were blinded to the clinical features of the patients, FBP, and other investigations. In case of discrepancy between the two reporting researchers, a third researcher (tie‐breaker) was used for reader agreement. The indicated link between PMN cell count and the 4‐grade scale (0, 1+, 2+, and 3+) of the strip was as follows: 0 = 0 PMN cells/mm^3^, 1+ = ≥ 25 PMN cells/mm^3^, 2 + = ≥ 75 PMN cells/mm^3^, 3 + = ≥ 500 PMN cells/mm^3^. When the dipstick recorded 1+, 2+, 3+ was considered to be positive, and the patient was labeled as having SBP, and such colorimetric scales were analyzed as separate categories. Negative dipstick tests were defined as negative results on the colorimetric scale. Unreadable strip results were handled by repeating the test using a fresh ascitic fluid sample and a new strip. Also, indeterminate results were handled by repeating the tests and avoiding exclusion bias.

### 2.6 Preparation of the Cytological Slides and Manual Counting of the PMN Cells From the Ascitic Fluid

For manual counting of the PMN cells, 10 mL of the ascitic fluid, which was collected and immediately placed into an EDTA‐containing tube to prevent clotting was inoculated into heparin containing tubes and thereafter the tubes were sent for cytological evaluation for quantification of PMN cells within 4 h from the time of collection. The ascitic fluid was stirred briskly to disperse the suspended cells followed by centrifugation at 1500 rpm for 10 min and the majority of the supernatant was discharged to concentrate the cells. Each sample was smeared on 3–4 microscopic glass slides by dropping 1–2 drops of the sediment. The prepared smears were immediately dipped in 95% ethanol for fixation for at least 15 min. Staining of the smears was done using Papanicolaou stain. Cytological examination of the stained smears was done by two independent experienced pathologists who were blinded of the results of LERS, as well as using duplicate readings, so as to enhance reliability. Reporting of the smears was done using Leica DM300 microscope fitted with a digital camera.

Counting of the PMN cells was done by using the chamber counting method, and ≥ 250 cells/mm^3^ was used as a diagnostic cut‐off for establishing positive and negative results for SBP [25]. A measured portion of the remaining fluid (pellet) (40 μL) was mixed with a diluent Turk's fluid of 800 μL. Turk's fluid contains acetic acid to lyse the red blood cells (RBCs), and is used gently shaken and used to fill the counting chamber. gentian violet, which stains the white blood cell (WBC) nuclei, and this helps to make them visible [26]. The cells are counted (40 × objective) in one of the nine large squares, and the number of white blood cells per cubic millimeter calculated. Another 10 mL sample of ascitic fluid is used for the PMN percentage determination (100 × objective), after centrifugation and May GrünwaldGiemsa staining. Globally, irrespective of culture, the PMN ≥ 250/mm^3^ in ascitic fluid is known as the best marker of peritonitis [27, 28].

### 2.7 Statistical Analysis

Data were analyzed using SPSS program 25.0. Data cleaning was performed before analysis by running crosstabs, frequency, and percentages to cross‐check for missing, duplications, and wrongly entered data. Continuous variables were summarized in mean ± standard deviation (SD) after testing for assumption of normality of the data using Shapiro–Wilk test. All continuous variables that rejected the normality test were expressed as median (interquartile range). Categorical variables were presented as absolute numbers and percentages. Binary logistic regression analysis was performed to determine the association between the independent variables and a positive result for SBP. Diagnostic accuracy, sensitivity, specificity, PPV, and NPV were determined after comparing the results at three colorimetric scales (+1, +2, and +3) with those of ascitic fluid cytological results at a cut‐off level of PMN cell count ≥ 250 cells/mm^3^ (bacterascites). The final diagnostic performances of LERS in this study were considered at +2 colorimetric scale as it was done in previous studies [16, 24] due to high diagnostic performance. This is because the +1 and +2 colorimetric scales showed low diagnostic accuracy, sensitivity, specificity, PPV, and NPV. This work was prepared in adherence to STARD guidance, including clearer reporting of missing data, indeterminate results, agreement between readers, and justification of the selected cut‐off [29].

## 3 Results

### 3.1 Selection Process of the Study Subjects for the Study

**Figure 1:**
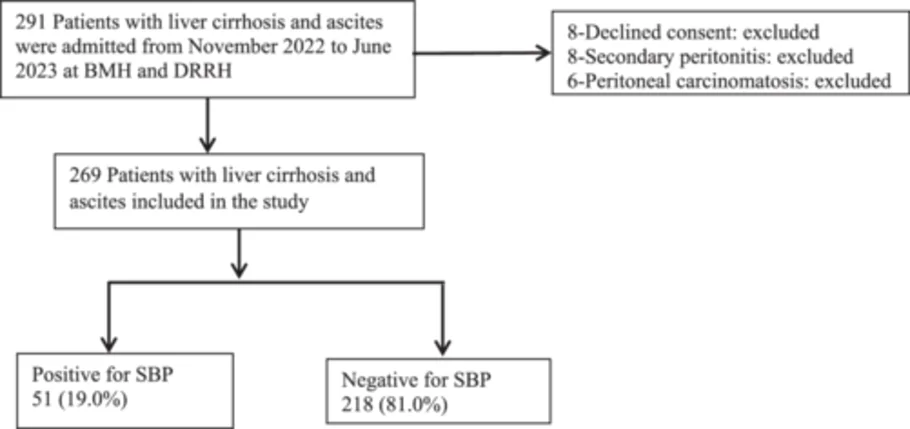
Flow chart showing selection process of the study subjects. The mean age of the patients was 46.17 ± 10.31 years. Majority (61.3%, *n* = 165) of the patients consisted of males, and over one‐third (39.8%, *n* = 107) of the patients were aged ≥ 60 years. Alcohol‐liver related disease accounted for over one‐third (44.2%, *n* = 119) of the contributing risk factors of liver cirrhosis among the patients followed by HBV infection, which was found in (29.4%, *n* = 79) of all the patients. Regarding clinical presentation of the patients, most of the patients (42.4%, *n* = 114) were presenting with upper gastrointestinal bleeding (UGIB). The frequency distribution of the various degree or grade of ascites among patients showed that, majority of the patients (69.5%, *n* = 187) had moderate (grade 2) degree of ascites. Also, over half of the patients (54.3%, *n* = 146) were in Child Pugh score class B (Table 1).

During the study period, a total of 291 patients with liver cirrhosis and ascites were admitted to the two study sites. A total of (7.6%, *n* = 22) patients were excluded from the analysis due to declining to sign written informed consent (8 cases), secondary peritonitis (8 cases), and peritoneal carcinomatosis (6 cases) (Figure 1). The retention rate of the study subjects in this study was (92.4%, *n* = 269), with (19.0%, *n* = 51) and (81.0%, *n* = 218) cases with and without SBP, respectively.

**Table 1:** Sociodemographic and clinical characteristics of the patients (*N* = 269).

Variables	Frequency (*n*)	Percentage (%)
Age (years)		
18–39	78	29.0
40–59	84	31.2
≥ 60	107	39.8
Sex		
Male	165	61.3
Female	104	38.7
Residence		
Rural	104	38.7
Urban	165	61.3
Comorbidities		
None	162	60.1
T2DM	44	16.4
Hypertension	51	19.0
HIV	12	4.5
Risk factors of liver cirrhosis		
Alcoholism	119	44.2
Hepatitis C	7	2.6
Hepatitis B	79	29.4
Alcoholism + HBV	48	17.8
Alcoholism + HCV	7	2.6
Others	9	3.3
Jaundice		
Yes	99	36.8
No	170	63.2
Abdominal pain		
Yes	100	37.2
No	169	62.8
Rebound tenderness		
Yes	31	11.5
No	238	88.5
Upper gastrointestinal bleeding		
Present	114	42.4
Absent	155	57.6
Hepatic encephalopathy		
Present	45	16.7
Absent	224	83.3
Ascites		
Mild	6	2.2
Moderate	187	69.5
Severe	76	28.3
Child Pugh score		
A	—	—
B	146	54.3
C	123	45.7

### 3.2 Laboratory Investigations of the Patients

The results of the laboratory investigations are presented in Table 2. The prevalence of SBP based on manual cell counting from the cytological smears prepared from the ascitic fluid using the cut‐off point of ≥ 250 PMN cells/mm^3^ was (19.0%, *n* = 51). Majority of the patients showed elevated AST and ALT which was observed in (87.7%, *n* = 236) and (88.8%, *n* = 239), respectively. Increased AST to platelet ratio index (APRI) score was seen in (66.9%, *n* = 180) of all the patients. This indicates that majority of the patients had fibrosis or rather severe liver disease due to APRI score > 1.5. Also, a total of (44.2%, *n* = 119) of the patients had raised level of model for end‐stage liver disease (MELD) score, this implies that almost half of the patients in the present study had end‐stage.

**Table 2:** Laboratory investigations and prevalence of spontaneous bacterial peritonitis (*N* = 269).

Variables	Frequency (*n*)	Percentage (%)
WBC (x 10^9^ cells/L)		
< 4.0	113	42.0
4.0–10.0	156	58.0
Hb (gm/dL)		
< 11.0	75	27.9
≥ 11.0	194	72.1
Platelets count (x 10^9^ cells/L)		
150–400	187	69.5
< 150	82	30.5
INR		
0.9–1.10	84	31.2
≥ 1.11	185	68.8
Albumin level (gm/L)		
< 3.2	142	52.8
≥ 3.2	127	47.2
Bilirubin (µmol/L)		
5.1–20.5	33	12.3
≥ 20.5	236	87.7
AST (U/L)		
8–31	30	11.2
≥ 31	239	88.8
ALT (U/L)		
7–34	100	37.2
≥ 34	169	62.8
AST/ALT ratio		
1–1.5	56	20.8
< 1.0	80	29.8
> 1.0	133	49.4
APRI score		
≤ 1.5	89	33.1
> 1.5	180	66.9
Creatinine (µmol/L)		
0–53	49	18.2
53.1–97.4	81	30.1
> 97.4	139	51.7
MELD score		
> 20	150	55.8
≤ 20	119	44.2
Spontaneous bacterial peritonitis		
Present	51	19.0
Absent	218	81.0

### 3.3 Diagnostic Performance of LERS for SBP Compared to Cytological Test Results as a Gold Standard Test

Tables 3–5 present 2 × 2 Tables indicating diagnostic indices at different colorimetric scales when compared to the results of the gold standard test which involved manual counting of the PMN cells from the ascetic fluid.

**Table 3:** Sensitivity, specificity, PPV, NPV, and diagnostic accuracy at colorimetric scale of 1+.

	Manual count of the PMN cells (Reference standard)			
Index test		Test + (≥ 250 PMN cells/mm^3^)	Test − (< 250 PMN cells/mm^3^)	Total
LERS test	Test +	36 (TP)	20 (FP)	56
	Test −	15 (FN)	198 (TN)	213
	Total	51	218	269

*Note:* TP‐True positive, FN‐False negative, FP‐False positive, TN‐True negative.

**Table 4:** Sensitivity, specificity, PPV, NPV, and diagnostic accuracy at colorimetric scale of 2+.

	Manual count of the PMN cells (Reference standard)			
Index test		Test + (≥ 250 PMN cells/mm^3^)	Test − (< 250 PMN cells/mm^3^)	Total
LERS test	Test +	41 (TP)	2 (FP)	43
	Test ‐	10 (FN)	216 (TN)	226
	Total	51	218	269

*Note:* TP‐True positive, FN‐False negative, FP‐False positive, TN‐True negative.

**Table 5:** Sensitivity, specificity, PPV, NPV, and diagnostic accuracy at colorimetric scale of 3+.

	Manual count of the PMN cells (Reference standard)			
Index test		Test + (≥ 250 PMN cells/mm^3^)	Test − (< 250 PMN cells/mm^3^)	Total
LERS test	Test +	32 (TP)	8 (FP)	40
	Test −	19 (FN)	210 (TN)	229
	Total	51	218	269

*Note:* TP‐True positive, FN‐False negative, FP‐False positive, TN‐True negative.

Tables 3 presents a 2 × 2 Table for the diagnostic indices at colorimetric scale 1 + . Sensitivity = 36/(36 + 15) × 100 = 70.6%, Specificity = 198/(20 + 198) × 100 = 90.8%, PPV = 36/(36 + 20) × 100 = 64.3%, NPV = 198/(15 + 198) × 100 = 93.0%, and diagnostic accuracy = (36 + 198)/269 × 100 = 87.0%.

Table 4 presents a 2 × 2 Table for the diagnostic indices at colorimetric scale 2+. Sensitivity = 41/(41 + 10) × 100 = 80.4%, Specificity = 216/(216 + 2) × 100 = 99.1%, PPV = 41/(41 + 2) × 100 = 95.3%, NPV = 216/(216 + 10) × 100 = 95.6%, and diagnostic accuracy = (41 + 218)/269 × 100 = 96.3%.

Table 5 presents a 2 × 2 Table for the diagnostic indices at colorimetric scale 3+. Sensitivity = 32/(32 + 19) × 100 = 62.7%, Specificity = 210/(210 + 8) × 100 = 96.3%, PPV = 8/(8 + 32) × 100 =20.0%, NPV = 210/(210 + 19) × 100 = 91.7%, and diagnostic accuracy = (32 + 210)/269 × 100 = 90.0%.

Table 6 presents a summary for the diagnostic indices at colorimetric scale 1+, 2+, and 3+. From the findings in this Table, it shows that at colorimetric scale of 2+ the diagnostic accuracy was high as it was marked by high sensitivity, specificity, and PPV compared to other categories (1+ and 2+) in which the diagnostic performance values were low. Therefore, colorimetric scale 2+ was considered the category comparable to the positive result of the reference standard (manual counting of ≥ 250 PMN cells/mm^3^ of ascetic fluid). Under this comparable point (colorimetric scale 2+), the sensitivity, specificity, PPV, NPV, and diagnostic accuracy were 80.4% (95% CI = 67.6–89.8), 99.1% (95% CI = 95.7–99.9), 95.3% (95% CI = 84.2–99.4), 95.6% (95% CI = 91.9–97.9), and 96.3% (95% CI = 92.3–97.6), respectively.

**Table 6:** Sensitivity, specificity, PPV, NPV, LRPT, LRNT, and 95% CI when we considered a reagent strip positive 1+, 2+, and 3+ colorimetric scales.

	Colorimetric scales (thresholds)		
	+1	+2	+3
Sensitivity	70.6% (95% CI = 56.2–82.5)	80.4% (95% CI = 67.6–89.8)	62.7% (95% CI = 48.1–75.4)
Specificity	90.8% (95% CI = 86.2–94.2)	99.1% (95% CI = 95.7–99.9)	96.3% (95% CI = 92.9–98.2)
PPV	64.3% (95% CI = 50.4–76.6)	95.3% (95% CI = 84.2–99.4)	20.0% (95% CI = 65.4–89.8)
NPV	93.0% (95% CI = 88.8–95.9)	95.6% (95% CI = 91.9–97.9)	91.7% (95% CI = 87.4–94.7)
DA	87.0% (95% CI = 82.4–90.7)	96.3% (95% CI = 92.3–97.6)	90.0% (95% CI = 85.8–92.9)
LRPT	7.7 (95% CI = 4.9–12.0)	87.6 (95% CI = 69.5.0–91.7)	17.1 (95% CI = 8.2–35.6)
LRNT	0.3 (95% CI = 0.2–0.5)	0.20 (95% CI = 0.11–0.4)	0.4 (95% CI = 0.3–0.6)

*Note:* PPV‐positive predictive value, NPV‐negative predictive value, LRPT‐likelihood ratio for a positive test, LRNT‐likelihood ratio for a negative test & DA‐diagnostic accuracy.

## 4 Discussion

This study aimed to investigate the prevalence of SBP and diagnostic performance of LERS by comparing with manual cell count of PMN cells from ascitic fluid smears as a gold standard diagnostic test for SBP in a cohort of cirrhotic patients with ascites at tertiary public health facilities. The study has shown a substantial prevalence of SBP. More importantly, LERS showed high diagnostic accuracy, which is crucial for early initiation of treatment of cirrhotic patients that had developed ascites.

The prevalence of SBP in this study was higher than 13.4% which was reported in the study of Zukerman et al which was done in Romania [30], however, close to 19.4% and 17.7% that was observed in the studies in India [24] and Cameroon [23], respectively. Moreover, higher prevalence of SBP of 27.4%, 26.6%, 26.4%, and 22.0% has been reported elsewhere [16, 31, 32, 33]. In another study of Elsadek et al which was done in Egypt reported significantly high positive result of SBP of 41.6% among cirrhotic patients with ascites [34]. Variation of accuracy of different brand names of LERS such as Siemens Multistix, Roche Chemtrip, Combur 10TestM, Combina 13 among many others also may help to explain the discrepancy in accuracy of the strips. There is a difference in the ability to detect presence of bacteria for these reagent strips, which contributes to the discrepancy of detection of SBP. Although there is a known cut‐off level of > 250 cells/mm^3^ based on the available guidelines [35], still there are challenges including lack of experience of performing manual cell count among reporters which contributes to subjectivity of the positive results. Currently, effort is being made to move from traditional manual cell count to automated cell counters like Beckman Coulter UniCel DxH 800 cellular analysis system [36]. This is because manual cell count approach has been found to be associated with operator dependency, transport‐induced cell lysis, and the potential for delays [37, 38]. Interobserver difference in reporting the prepared cytological smears from the ascitic fluid under microscopy also contributes to variation of results when counting manual counting of the cells.

Despite challenges that normally affect the diagnostic performance of LERS in diagnosing SBP among cirrhotic patients with ascites, the diagnostic accuracy of this test is quite high which ranges from 40% to 90% or over [17]. This is valuable for early treatment of the patients, and this helps to reduce morbidity and mortality. The accuracy of LERS in diagnosing SBP in our study of 95.9% considered at colorimetric scale 2+ was close to 96.1%, 94.5%, and 90.7% which were reported previously [16, 24, 31]. However, slightly lower levels of accuracy compared to the accuracy in this study of 86.3% and 89% were reported in the studies of Allam et al [38] and Rerknimitr et al [39], respectively. Various factors have been reported to be attributable to reduced diagnostic accuracy of LERS such as reduced number of PMNs in the ascitic fluid, challenges with urine‐specific calibration, and subjective interpretation of the colorimetric reaction [40]. Alteration of PH, osmolality and temperature of the specimens have been associated with false positive results [41]. Furthermore, sometimes non‐leucocyte cells can release esterase from certain organs like pancreas and liver, and this usually contributes to false positive results [42, 43]. Despite the observed diagnostic accuracy of LERS, still such tests have limited diagnostic accuracy for the diagnosis of SBP, and for that reason they should be used as surrogate test especially, in areas where there is a challenge of going bacterial culture test which is the gold standard for diagnosis of SBP [27].

This study demonstrated high sensitivity and specificity at the selected cut‐off value of +2 colorimetric scale close to the findings in the study of Chugh et al who used Multistix 10 SG reagent strip in diagnosing SBP in which the sensitivity and specificity was 95% and 96.4% at a cut‐off of grade +2, respectively [24]. However, in a study of Afrida et al which used Combur10 Test®M to assess the diagnostic performance of LERS in diagnosing SBP at a threshold level +2 colorimetric scale though the study revealed a high specificity of 94% and sensitivity of 63.9% [44]. A significantly very low sensitivity of 45.3% despite high specificity of 99.2% was reported in the study of Nousbaum et al in which Multistix 8SG urine strips for LERS were used for detection of SBP at +2 grade scale [45]. Difference of PMN count cut‐off levels for ascitic fluid vary from that of urine which is the preliminary purpose of test for these strips, difference in diagnostic performance between brand of strips, and experience of technicians while taking readings which can bring interobserver variation due to difference in grading for each strip, that is why it is necessary to use the cut‐off leucocyte count so as to make logical inferences [27].

Another important diagnostic performance of LERS is their high NPV which helps them to discriminate truly individuals without disease from the ones with the disease [46] by ensuring that there is very low rate of false‐negatives [47]. This helps clinicians to apply LERS as a possible adjunctive screening tool rather than a definitive substitute for standard diagnostic assessment. This may help to prevent delay of treatment or missed diagnosis, thereby ensuring timely and effective patient care. The majority of studies have shown very high NPV of various types or brands of LERS similar to the one which was observed in our study. Other studies have even shown NPV of 100%, indicating that in those studies there were no false‐negative results [48, 49]. Furthermore, in most studies LERS have demonstrated high PPV, this shows that these screening tests are valuable in detecting cases with SBP from the population of cirrhotic patients. The strip of LERS which was used in this study (CYBOWTM series) showed very high PPV similar to most of studies published previously [50, 51].

### 4.1 Study Limitations

The study limitations for this study included failure to including culture test for the patients due to lack of funds, and the subjectivity of the reading strips. Selection bias could also have influenced the findings due to convenience sampling of the study participants. Generalizability of the findings of this study was also limited due to small sample size. Other limitations included absence of ascitic fluid culture, and therefore we acknowledge that bacterascites and culture‐negative neutrocytic ascites could not be distinguished.

## 5 Conclusion

This study has shown a relatively significant number of the patients in the study had SBP. More importantly, our study has revealed high diagnostic performance for SBP due to high diagnostic accuracy, sensitivity, specificity, PPV, and NPV which were observed. Therefore, based on our findings and experience from previous data, it is evidently that LERS should be presented as a possible adjunctive screening tool, not as a definitive diagnostic replacement especially, in remote areas of resource‐limited settings where diagnostic tests like culture are still challenging. This is because a sensitivity of around 80% of LERS for the diagnosis of SBP would still miss a clinically important proportion of the SBP cases.

## Author Contributions

**Salum Ali Mwinyi:** conceptualization, investigation, writing – original draft, writing – review and editing. **Emmanuel Marcel Sindato:** validation, visualization, project administration, supervision, formal analysis, writing – review and editing, writing – original draft. **Emmanuel Muhimbura:** validation, visualization, writing – review and editing, formal analysis. **Gerald Jacob Kawishe:** visualization, validation, writing – review and editing, investigation. **James Joseph Yahaya:** writing – review and editing, writing – original draft, investigation, software.

## Funding

The authors have nothing to report.

## Ethics Statement

Ethical approval was obtained from the Research Ethical Committee of the University of Dodoma (reference: MA.84/261/02/10 & date: 02nd November 2022). We also confirm that all methods were performed in accordance with the relevant guidelines and regulations. All study participants signed written informed consent and confidentiality was maintained by anonymizing the identities of the participants including ensuring voluntariness, and compensation where necessary.

## Consent

The authors have nothing to report.

## Conflicts of Interest

The authors declare no conflicts of interest.

## Transparency Statement

The author, James Joseph Yahaya, affirms that this manuscript is an honest, accurate, and transparent account of the study being reported; that no important aspects of the study have been omitted; and that any discrepancies from the study as planned (and, if relevant, registered) have been explained.

## Data Availability

The dataset used to generate the findings of this study is restricted as per Ethical Research Committee of the University of Dodoma. However, data may be made available by upon a reasonable request from the corresponding author.
